# Advances in autophagy for Parkinson’s disease pathogenesis and treatment

**DOI:** 10.20517/and.2024.33

**Published:** 2025-03-17

**Authors:** Xiaojie Zhang, Huan Zhang, Jie Dong, Huaibin Cai, Weidong Le

**Affiliations:** 1 Transgenics Section, Laboratory of Neurogenetics, National Institute on Aging, National Institutes of Health, Bethesda, MD 20892, USA.; 2 Department of Neurology, Shanghai Jiao Tong University School of Medicine Affiliated Sixth People’s Hospital, Shanghai 200233, China.; 3 Department of Ultrasonography, Shanghai Jiao Tong University School of Medicine Affiliated Sixth People’s Hospital, Shanghai 200233, China.; 4 Shanghai University of Medicine & Health Sciences Affiliated Zhoupu Hospital, Shanghai 201318, China.

**Keywords:** Parkinson’s disease, autophagy, chaperone-mediated autophagy, mitophagy, lysosome

## Abstract

Autophagy is a cellular process essential for maintaining neuronal homeostasis by degrading and recycling damaged organelles and proteins. Impairments in canonical autophagy pathways, such as macroautophagy, chaperone-mediated autophagy (CMA), and mitophagy, are linked to Parkinson’s disease (PD) pathogenesis, contributing to α-synuclein aggregation and dopaminergic neuronal loss. Moreover, the recent discovery of noncanonical autophagy highlights the unexpected roles of autophagy-related proteins in protein degradation beyond the canonical autophagy pathways. Advances in understanding the molecular mechanisms of autophagy provide potential therapeutic strategies to modulate this pathway in PD. Key therapeutic targets include mTOR and AMPK, with compounds like rapamycin, trehalose, and resveratrol showing promise in preclinical models. Enhancing lysosomal function and mitophagy also presents a viable strategy to alleviate PD symptoms. This review emphasizes the complex roles of autophagy in PD and highlights the potential of autophagy modulation as a promising therapeutic strategy for treating the disease.

## INTRODUCTION

Parkinson’s disease (PD) is one of the most prevalent neurodegenerative disorders, with an estimated 13 million individuals expected to get a diagnosis worldwide by 2040^[1]^. Individuals with PD display a broad range of motor symptoms^[2]^, such as akinesia/bradykinesia, resting tremor, and rigidity, and non-motor symptoms^[3]^, including cognitive impairment, sleep disorders, chronic pain, and autonomic dysfunction. PD is pathologically defined by the progressive degeneration of dopaminergic neurons in the substantia nigra pars compacta (SNc), along with the formation of intracytoplasmic inclusions termed Lewy bodies (LBs), predominantly composed of fibrillar α-synuclein (α-syn) aggregates^[4,5]^. Using advanced tools to detect dysfunctional α-syn in patients, recent studies have redefined this group of diseases as α-synucleinopathies, including PD^[6]^. Strategies targeting misfolded and aggregated α-syn hold promise for more effective treatments for PD.

Autophagy is a conserved process that maintains cellular homeostasis by degrading misfolded proteins, excessive organelles, and pathogens. Numerous studies have shown that alterations in autophagy are associated with neurodegenerative diseases, including PD^[7,8]^. Genetic studies, particularly genome-wide association studies (GWAS) have observed several autophagy-associated genes as risk factors in PD, including α-syn-encoding gene (*SNCA*), leucine-rich repeat sequence kinase 2 (*LRRK2*), Parkin RBR E3 ubiquitin-protein ligase (*PARK2*, *Parkin*), Parkinson protein 7 (*PARK7*, *DJ-1*), and PTEN-induced kinase 1 (*PINK1*)^[9]^. Autophagic dysfunction has been identified in the brain tissue of post-mortem PD individuals, suggesting a connection to α-syn pathology^[10]^.

This review explores how proteins linked to PD affect autophagy and how autophagy dysfunction impacts the accumulation of α-syn aggregates. Additionally, we summarize recent advances in developing autophagy-related therapeutics for PD.

## AUTOPHAGY IN THE PATHOGENESIS OF PD

### Canonical autophagy

Most neurodegenerative diseases are linked to the aberrant accumulation of neurotoxic protein aggregates, which disrupt the protein degradation pathway and lead to neuronal degeneration^[8,11]^. Autophagy is a predominant degradation and recycling process that sustains the physiological balance of neurons and glial cells in the central nervous system^[12]^. Classical or canonical autophagy in mammalian cells is categorized into three specific routes according to the type of cargo delivered to the lysosome: (1) macroautophagy; (2) microautophagy; and (3) chaperone-mediated autophagy (CMA). Microautophagy will not be discussed in this review as fewer studies are involved in this pathway in PD.

In macroautophagy (hereafter as autophagy), the cytoplasm material is sequestered into double-membraned autophagosomes delivered to lysosomes for degradation^[13]^. Autophagy is considered a nonselective process, but selective autophagy requires the identification of specific cytoplasmic cargo through autophagy receptors, with mitophagy being the most critical form^[14]^. CMA is a highly selective process that degrades specific substrates containing a recognition sequence (KFERQ-like peptide motif) via the action of the heat shock cognate 70-kDa protein (HSC70)^[15]^. The substrates of all the above three autophagic pathways are eventually degraded in lysosomes under the action of serial different autophagy-related proteins in induction/nucleation, elongation, delivery, fusion with lysosome, degradation, and recycling^[16]^.

**Figure 1.**
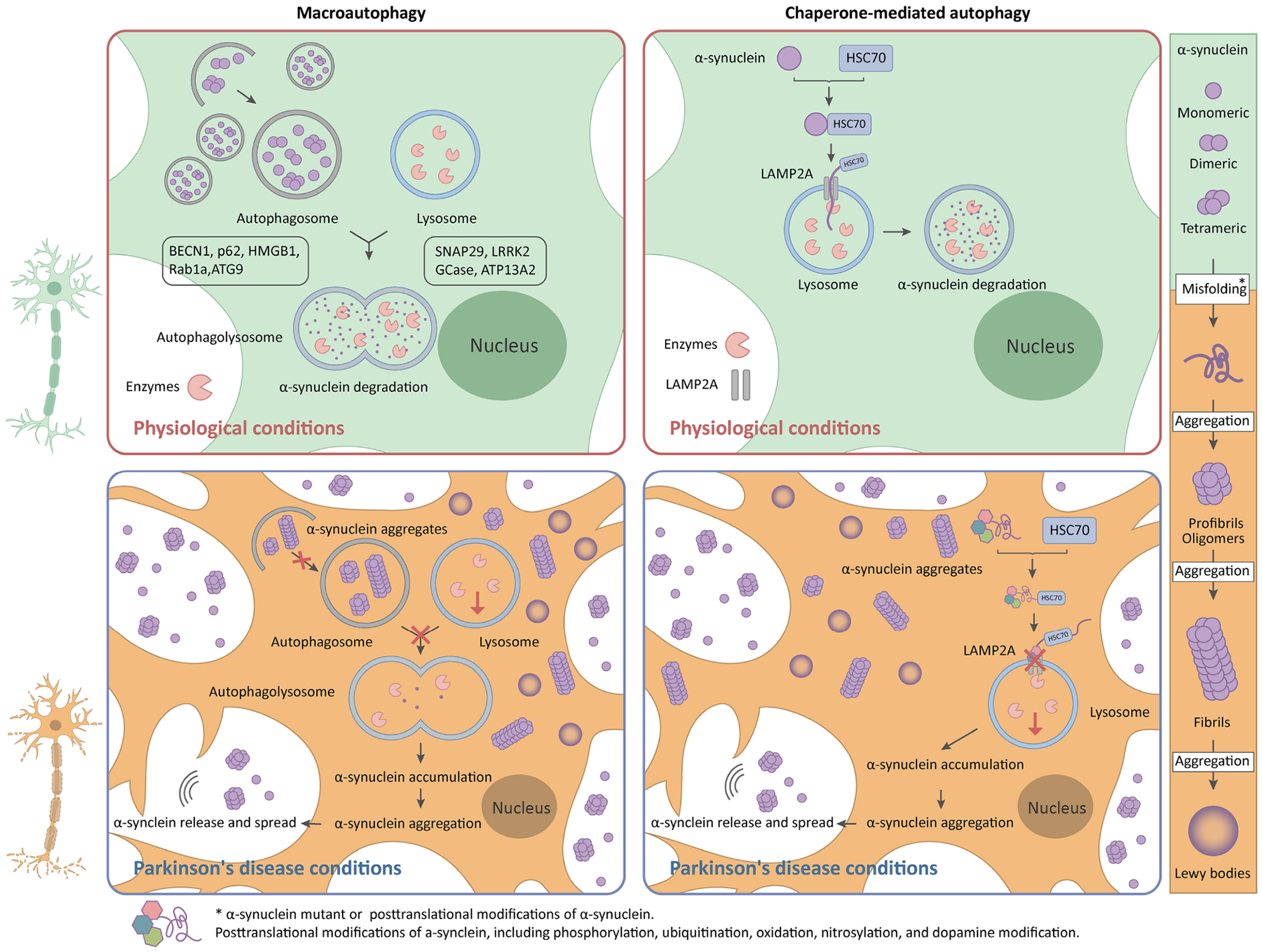
Schematic illustration of PD-related genes involved in different steps of autophagy. Many genes linked to PD are associated with macroautophagy, CMA, mitophagy, and subsequent lysosomal functions, which are listed in the white boxes of the scheme. Macroautophagy is initiated by a phagophore, forming the double-membraned autophagosome to sequester cytosolic constituents and damaged organelles. Then, the fusion of autophagosomes with lysosomes forms the autolysosome for degradation of its contents. In CMA, the chaperone protein HSC70 targets and transports unfolded proteins to lysosomes by binding to the lysosomal receptor LAMP2A. Selective mitophagy labels only damaged mitochondria for downstream autophagic degradation. In addition, VPS35 plays an important role in the trafficking between endosomes and the trans-Golgi network. PD: Parkinson’s disease; CMA: chaperone-mediated autophagy; HSC70: heat shock cognate 70-kDa protein; LAMP2A: lysosome-associated membrane protein 2A; VPS35: vacuolar protein sorting 35.

Deficiency at different stages of autophagy may promote toxic protein aggregation (α-syn) and exacerbate neuron degeneration, thus contributing to PD pathogenesis. Conversely, PD-associated mutations can also impair different steps of autophagy [Figure 1], forming a bidirectional loop.

Genetic mutations in *LRRK2* account for many autosomal dominant cases of PD^[17]^. LKKR2 is a multifunctional serine-threonine kinase that participates in various cellular signaling pathways, such as autophagy, vesicular trafficking, and neurite growth^[18]^. Pathogenic mutations of *LRRK2*, such as G2019S, enhance kinase activity^[19]^. LRRK2 phosphorylates Endophilin-A to induce autophagosome formation, thus initiating autophagy induction in the presynaptic terminal^[20]^. Autophagy in *LRRK2* G2019S mutations is mediated by the mitogen-activated protein kinase/extracellular signal-regulated protein kinase (MAPK/ERK) pathway^[21]^. Moreover, LRRK2 modulates lysosomal function through its kinase activity on several Rab GTPases^[22]^. Overexpression of LRRK2 interferes with Rab phosphorylation and results in lysosome defects^[23]^. Mutations in LRRK2 enhanced HSC70 and lysosome-associated membrane protein 2A (LAMP2A)^[24]^. Additionally, they increased the interaction between α-syn and LAMP2A to prevent α-syn from translocating into lysosome from being degraded, leading to an elevation of α-syn aggregation^[24]^. Furthermore, *LRRK2* G2019S variations impaired PINK1/Parkin-dependent mitophagy by increasing mitochondrial aggregation and attenuating mitochondrial clearance^[25]^. In summary, LRRK2 pathogenic mutations lead to the impairment of autophagy, influence lysosomal function, and block CMA, which accelerates the progress of PD.

#### PINK1 and PRKN (PARK2, Parkin)

Mutations in *PINK1* and *PRKN* (*PARK2*, *Parkin*) are recognized as the predominant cause of early-onset PD^[26,27]^. PINK1 and Parkin are functionally interrelated and regulate the mitochondrial quality control pathway together^[28]^. Under mitochondrial stress, homodimerization of PINK1 is promptly located on the outer membrane of impaired mitochondria, leading to autophosphorylation, which induces kinase activation and facilitates binding to substrates Parkin and ubiquitin^[29]^. Activated PINK1 phosphorylates Parkin on its ubiquitin-like domain, increasing Parkin’s E3 ubiquitin ligase activity^[30]^. PINK1 and Parkin marked damaged mitochondria in response to stress by labeling phosphorylated poly-Ub (p-S65-Ub) chains^[31]^. These specific tags are then recognized by autophagy receptors, leading to degradation by mitophagy. *PINK1* or *PRKN* loss of function mutations inhibit mitophagy and, subsequently, result in the accumulation of impaired mitochondria and potentially lead to neuronal death^[32]^. Dopaminergic neurons are particularly vulnerable to mitochondrial dysfunction and oxidative stress; thus, mutations in PINK1 or PRKN may lead to selective neurodegeneration in the area of SNc in PD^[33]^.

#### The glucosylceramidase beta

The glucosylceramidase beta (*GBA*) gene encodes the lysosomal glucocerebrosidase enzyme (GCase), which regulates glycosphingolipid homeostasis^[34]^. Homozygous mutations in the *GBA* gene result in Gaucher disease, the most prevalent lysosomal storage disease. Numerous cohort studies have investigated the correlation between *GBA* mutations and the likelihood of PD. Approximately 5%−15% of PD individuals carry *GBA* mutations, giving it a significant genetic risk factor for the disease^[35]^. Mutations in the *GBA* gene can diminish GCase activity and lysosomal dysfunction, hindering protein degradation, particularly the α-syn metabolism. The functional loss of GCase due to PD-related *GBA* mutations caused the accumulation of α-syn, autophagic-lysosomal pathway dysfunction, CMA impairment, and lipid metabolism abnormality^[36]^. PD patient-derived fibroblasts with GBA mutations show compromised autophagy lysosomal reformation, decreased phospho-S6K, and accumulated Rab7, which is a GTPase critical for endosome-lysosome trafficking, supporting the notion that lysosomal recycling is impaired^[37]^. Furthermore, mutations in GBA may result in misfolded GCase being retained in the endoplasmic reticulum (ER), hence inducing ER stress, which may contribute to neurodegeneration in PD^[38]^. Moreover, a deficit in GCase has been linked to mitochondrial malfunction and mitophagy. Autophagy-mediated removal of damaged mitochondria was impaired in iPSC-derived dopamine neurons from carriers of *GBA* mutations^[39]^. The accumulation of pathogenic α-syn can increase the vulnerability of dopamine neurons to mitochondrial impairment induced by 1-methyl-4-phenyl-1,2,3,6-tetrahydropyridine (MPTP) in a mouse model with GBA mutation^[40]^, implicating the link between GBA, α-syn, and mitochondrial dysfunction.

#### ATPase cation transporting 13A2

Mutations in ATPase cation transporting 13A2 (ATP13A2) result in an early-onset variant of PD. ATP13A2 acts as a lysosomal type 5 P-type ATPase, contributing to lysosome acidification and α-syn degradation^[41]^. ATP13A2 mutations function as a negative regulator of the autophagy pathway, inhibit lysosomal function in dopaminergic neurons, and lead to α-syn aggregation and neuron death. Mutations in ATP13A2 act as inhibitors of the process of autophagy. The deficiency of lysosomal activity in dopaminergic neurons results in the aggregation of α-syn and neurotoxicity^[42]^.

#### DJ-1

The *DJ-1* (*PARK1*) gene mutation causes early-onset autosomal recessive PD. The DJ-1 protein is present in the cytosol, nucleus, and mitochondria and plays key roles in mitochondrial function, protein synthesis, regulation of the proteasome, and chaperone activities^[43]^. Wild-type DJ-1 can act as an oxidative stress sensor to inhibit α-syn aggregation and its oxidation to prevent α-syn fibrillation^[44]^. Mutations in DJ-1 linked to PD enhance α-syn aggregation in dopaminergic neurons and glial cells. DJ-1 dysfunction enhanced the LAMP2A degradation in lysosomes, resulting in α-syn accumulation by blocking the CMA pathway^[45]^. Furthermore, DJ-1 acts downstream of PINK1 mitophagy to maintain mitochondrial quality control^[46]^.

#### Vacuolar protein sorting 35

VPS35 mutations are linked to late-onset autosomal dominant PD^[47]^. VPS35 is an essential part of the retromer cargo-recognition complex that recycles membrane proteins between endosomes and the trans-Golgi network. The expression of D620N VPS35, the predominant mutation associated with PD, resulted in irregular trafficking of the autophagy protein ATG9A and impaired autophagosome formation^[48]^. In addition, it has been proposed that VPS35 deficiency or mutation decreases the degradation of α-syn and contributes to the pathogenesis of PD by impairing CMA in dopaminergic neurons, primarily by limiting the retrieval of LAMP2A from endosomes to the Golgi^[49]^.

#### Other genes

Some other genes associated with PD pathogenesis, such as F-box protein 7 (*FBXO7*) and vacuolar protein sorting 13 homolog C (*VPS13C*), also regulate autophagic functions. The proteins encoded by these genes contribute to mitophagy^[50,51]^. FBXO7 contributes to mitochondrial maintenance by directly interacting with PINK1 and PARK and is involved in Parkin-mediated mitophagy. Mutations or deficiencies in FBXO7 block the translocation of Parkin to mitochondria, thereby impairing mitophagy^[50]^. In addition, *VPS31C* mutations are related to a distinct form of early-onset Parkinsonism. VPS13C localized to the outer membrane of mitochondria. The deficiency of VPS13C interacted with lower mitochondrial membrane potential, mitochondrial fragmentation, and PINK1/Parkin-dependent mitophagy^[51]^.

### Noncanonical autophagy

Recent research suggests autophagy factors are involved in processes apart from canonical autophagy. The autophagy-related mechanisms known as noncanonical autophagy or the conjugation of ATG8 to single membranes (CASM) are characterized by the generation of single-membrane vesicles inside the endolysosomal system, facilitating ATG8/LC3 conjugation and subsequent lysosomal destruction^[52]^. Multiple mechanisms enhanced the comprehension of noncanonical autophagy: LC3-associated phagocytosis (LAP), LC3-associated endocytosis (LANDO)^[53]^, endosomal microautophagy (eMI)^[54]^, and LC3-dependent extracellular vesicle (EV) loading and secretion (LDELS)/secretory autophagy (SA)^[55]^.

The exploration of the specific role of noncanonical autophagy in PD remains insufficient despite relevant evidence suggesting the involvement of SA in the degradation of abnormally aggregated α-syn. Previous studies have shown that α-syn is released from neurons in models of PD^[56]^. This release may indicate a compensatory mechanism to impaired degradative autophagy, potentially shifting to SA to preserve proteostasis in neurons, similar to mitochondrial SA observed in the model of cardiomyocytes^[57]^. More evidence is needed to demonstrate the possible mechanisms of noncanonical autophagy in PD. Eliminating extracellular α-syn via microglial autophagy is proposed as essential for preserving neuronal protection and function. Canonical and noncanonical autophagy pathways are believed to be essential for the functioning of microglia, likely operating in complementary manners^[58]^. This begs whether microglia-related noncanonical autophagy can protect neuronal survival in PD by removing aggregated α-syn proteins.

## AUTOPHAGY AND α-SYN

α-syn, which is encoded by the *SNCA* gene, is a 14 KDa presynaptic protein mainly expressed in brain neurons and other tissues such as the cerebrospinal fluid, blood, and plasma^[59]^. In the physiological state, endogenous α-syn monomers naturally form dimers and tetramers with little or no aggregation potential^[60]^. α-syn shows a pathological conformation in PD that recruits more monomers to form oligomers and fibrils in the axons or soma of dopaminergic neurons to comprise LBs^[5,61]^. Abnormally aggregated α-syn can be degraded via multiple clearance pathways, including autophagy and CMA. Additionally, unlike preformed α-syn fibrils, a unique nonfibrillar and highly neurotoxic α-syn species, termed “pα-syn*”, was identified in primary neurons, mouse brains, and the brains of PD individuals^[62]^. pα-syn* arises from the insufficient autophagic degradation of preformed α-syn fibrils. pα-syn* colocalized with Bip, an essential regulator of the unfolded protein response and a resident protein of mitochondria-associated ER membranes, inducing mitochondrial toxicity, energetic stress, and mitophagy^[62]^.

**Figure 2.**
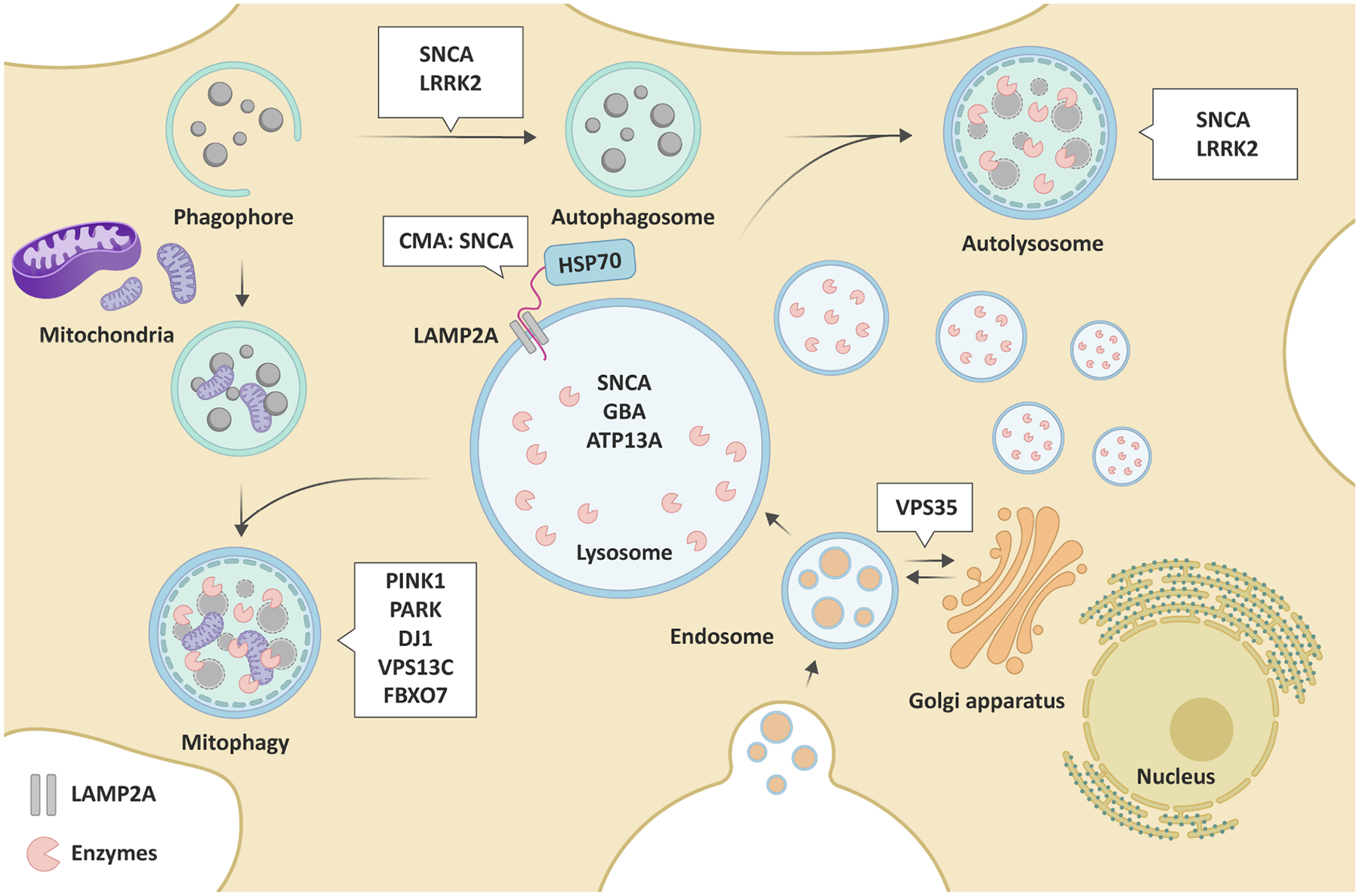
Schematic of the roles of α-syn in autophagy in PD. In the physiological state, macroautophagy and CMA pathways are involved in α-syn degradation (monomeric, dimeric, and tetrameric forms). In pathological conditions of PD, *SNCA* mutations contribute to the pathological aggregation of α-syn, such as oligomer, fibrosis, and Lewy body forms, which leads to impairments of autophagy and CMA pathways. Pathological aggregated α-syn interferes with autophagosomal formation, blocks the fusion of autophagosomes with lysosomes, and impairs the lysosomal activity. Additionally, mutants or other posttranslational modifications of α-syn bind with LAMP2A with high affinity, blocking the degradation of other substrates in the CMA pathway and resulting in lysosomal dysfunction. Impaired autophagy and CMA further exacerbate the aggregation of pathological α-syn, subsequently leading to the death of dopaminergic neurons. Furthermore, under conditions of lysosomal dysfunction, autophagic impairment, and proteasome inhibition, pathological α-syn serves as a seed that spreads to surrounding neurons, leading to more neuronal death. α-syn: α-synuclein; PD: Parkinson’s disease; CMA: chaperone-mediated autophagy; SNCA: α-syn-encoding gene; LAMP2A: lysosome-associated membrane protein 2A.

There is much evidence of a close relationship between α-syn and the autophagy pathway. Figure 2 shows a schematic of the roles of α-syn in autophagy under physiological and PD conditions.

Firstly, mutant α-syn can lead to autophagy upregulation marked by elevated levels of LC3-II and BECN1, a compensatory mechanism for degrading pathological forms of α-syn by the proteasome pathway^[63]^. In addition, the expression of the A53T mutant, as opposed to wild-type α-syn in PC12 cells, leads to autophagosome impairment, lysosomal hydrolysis deficiency, proteasomal dysfunction, reduced dopamine release, and autophagic cell death^[64]^. Secondly, α-syn impaired the cytosolic translocation of nuclear high mobility group box 1(HMGB1), inhibited the interaction between HMGB1 and Beclin 1 (BECN1), and enhanced the binding of BECN1 to Bcl2, thus disrupting the autophagy process^[65]^. Furthermore, overexpression of wild-type α-syn impairs autophagy via Rab1a inhibition, a GTPase that plays a crucial role in the initial step of autophagosome synthesis, and results in the mislocation of ATG9 and decreases the formation of omegasomes, the autophagosome precursors^[66]^. Finally, overexpression of α-syn inhibits autophagy by preventing SNAP29-mediated autophagosome-lysosome fusion, which is induced by an increase in the number of EVs and autophagy-associated proteins in these EVs^[67]^. Taken together, mutant α-syn can stimulate autophagy, block the formation of autophagosomes, impair the process of autophagosome-lysosome fusion, and influence lysosomal function.

The structure of α-syn contains a motif for binding the HSC70, VKKDQ (a KFERQ-like motif). This motif allows it to bind to LAMP2A at the lysosomal membrane, leading to its degradation via the CMA pathway^[68]^. Generally, monomers of α-syn are preferred to be degraded by CMA, while autophagy has a critical role in degrading aggregates or fibrosis^[69]^. However, pathogenetic α-syn mutants seem to block the pathway of CMA. A53T and A30P mutants of α-syn bind with LAMP2A with high affinity, impairing the uptake and degradation of other substrates in the CMA pathway and inducing the abnormal activation of autophagy as a compensatory mechanism^[70]^. Besides pathogenic PD mutations, posttranslational modifications of α-syn, including phosphorylation, ubiquitination, oxidation, nitrosylation, and dopamine modification, have been shown to block its CMA-mediated degradation and prompt abnormal protein aggregation^[71,72]^. Dopamine-modified α-syn induces severe blockage of CMA, implying the selective vulnerability of neurons in the SNc in PD^[71]^. Furthermore, CMA blockage by mutant α-syn leads to dominant α-syn accumulation in the brainstem and spinal cord, which have lower CMA activity than other brain regions^[44]^. These results indicate potential mechanisms that elucidate the vulnerability or resistance of specific brain regions in the pathogenesis of PD.

The transmission of α-syn between neurons occurs through endocytosis, plasma membrane penetration, or exosomes^[73]^. Consequently, it propagates Lewy body pathology across various brain regions, thereby contributing to the progressiveness of PD. It is secreted in response to multiple stressors, such as oxidative stress, lysosomal dysfunction, proteasome inhibition, and autophagic impairment^[73,74]^. α-syn synthesis, aggregation, uptake by neighboring cells, and degradation either inside or outside cells will influence the extent of affected neurons^[56]^. Thereby, the spreading of α-syn within the dopaminergic neurons has been proposed as one of the mechanisms associated with the progression of PD.

## AUTOPHAGY-BASED THERAPEUTIC STRATEGIES FOR PD

Table 1 summarizes potential therapeutic opportunities in different targeting options, such as initiating autophagy, mitophagy, lysosomes, LRRK2 inhibition, and microRNA (miRNA) targeting autophagy in PD.

### Promoting autophagic initiation

The therapeutic strategies promoting autophagic initiation for PD treatment focus on modulating upstream regulators of the autophagy pathway by mTOR-dependent and -independent signaling. Rapamycin and its analog, temsirolimus, inhibit mTORC1 and then activate autophagy. These agents enhance α-syn clearance and protect dopaminergic neuron survival in animal models, though long-term or high-dose use raises side effects such as immunosuppression risks^[75,76]^. Metformin and resveratrol are AMPK activators that enhance autophagy and protect dopaminergic neurons^[77,78]^. PREP inhibitor (KYP-2047) and isorhynchophylline enhance α-syn clearance by activating Beclin-1 and initiating the autophagic process in PD models^[79,80]^. Furthermore, some agents bypass mTOR to activate autophagy through other pathways. Lithium can induce autophagy in an mTOR-independent manner by directly inhibiting IMPase, which has shown efficacy in reducing α-syn accumulation and preventing mitochondrial dysfunction^[81]^. Trehalose, a disaccharide, suppresses the activity of SLC2A (family of glucose transporters), enhances mTOR-independent activation of autophagy, and promotes α-syn clearance^[82]^. However, these compounds can affect multiple cellular pathways and numerous mechanisms of action. Balancing efficacy and minimizing side effects remain critical challenges for clinical applications.

### Enhancing lysosomal function

Lysosomal dysfunction is a hallmark of PD, and mutations in genes like ATP13A2 and GBA impair lysosomal acidification and reduce the clearance of autophagosomes, leading to the degeneration of dopaminergic neurons. Enhancing lysosomal function can potentially rescue autophagy and alleviate PD symptoms. Rifampicin and clioquinol are promising candidates for restoring lysosomal acidification^[83,84]^, while compounds like ML-SA1 can increase lysosomal biogenesis and function^[85]^. All these compounds show the effects of ameliorating autophagic flux and reducing α-syn accumulation by restoring lysosomal function. Other pharmacological approaches aim to block c-Abl, a kinase involved in the later stages of the autophagy pathway, by modulating lysosomal maturation through enzymes such as cathepsin D and cathepsin L. The c-Abl modulator, Nilotinib, showed favorable tolerability and safety but no improvement in the motor outcome of PD patients^[86]^.

Furthermore, the transcription factor EB (TFEB) has been considered a critical regulator of lysosomal biogenesis and function. Agents like 2-Hydroxypropyl-β-cyclodextrin chemically activate TFEB, enhancing lysosomal function and promoting autophagic clearance of α-syn^[87]^. Mutations in GBA1 result in diminished GCase activity and subsequent lysosomal dysfunction. Chaperones like ambroxol and isofagomine stabilize GCase, enhance its trafficking, and restore lysosomal function in PD models^[88]^. Recent studies indicate that acidic nanoparticles may enhance lysosomal degradation by decreasing lysosomal pH, thereby restoring lysosomal function in various PD models, including those with ATP13A2 and GBA mutations^[89]^.

### Targeting mitophagy

Dopaminergic neuron loss in PD is closely associated with mitochondrial dysfunction, as these cells require substantial energy and are thus highly susceptible to mitochondrial damage^[90]^. The PINK1/Parkin-mediated mitophagy pathway and alternative pathways regulated by BNIP3L/Nix and FUNDC1 are gaining attention as potential targets for enhancing mitophagy in treating PD^[91]^.

Research aimed at improving PINK1/Parkin mitophagy has concentrated on small molecules to increase the activity of PINK1/Parkin. Kinetin triphosphate (KTP), which mimics ATP, enhances PINK1 activity and PINK1-mediate mitophagy^[92]^. Drugs like niclosamide induce mitophagy by causing mild mitochondrial depolarization, thus indirectly activating PINK1^[93]^. USP30 inhibitors, such as FT3967385 and MF-094, induce mitophagy by blocking the deubiquitination process that opposes Parkin, thereby promoting the removal of damaged mitochondria^[94]^. Otherwise, BNIP3L/Nix and FUN14 domain-containing protein 1 (FUNDC1) pathways can promote mitochondrial degradation independently of PINK1 and Parkin, contributing to resistant mitochondrial dysfunction. BNIP3L/Nix is activated under hypoxia, while FUNDC1 responds to cellular stress, recruiting LC3 to initiate mitophagy^[95,96]^.

Furthermore, autophagy-targeting chimeras (AUTACs) represent a novel strategy that directly bypasses traditional pathways to induce mitophagy with small molecules. Iron chelation therapy with agents like deferiprone also induces mitophagy independently of PINK1/Parkin, which has shown preliminary success in clinical trials for PD^[97,98]^.

### Regulating autophagy by miRNAs

miRNAs are small, non-coding RNAs that regulate gene expression by targeting mRNA, influencing various cellular functions, including autophagy. Recent studies have indicated that specific miRNAs can regulate autophagy-related genes and act as neuroprotective effectors by activating autophagy, clearing α-syn aggregates and damaged mitochondria, and then alleviating the disease progress of PD^[99]^. For instance, miRNAs like miR-326 and miR-124 have been shown to reduce α-syn levels and support dopaminergic neuron survival in PD models by enhancing autophagy^[99,100]^. Alternatively, other miRNAs are upregulated in PD, implicating disease exacerbation by inhibiting autophagy, such as miR-204–5p^[101]^ and miR-19a-3p^[102]^. These findings indicate that targeting autophagy-regulating miRNAs could offer new therapeutic strategies for PD.

Strategies include modulating miRNA levels using long non-coding RNAs (lncRNAs), circular RNAs (circRNAs), and natural agents to restore miRNA-mediated autophagy in PD models and patients. OIP5-AS1, a lncRNA targeting miR-137, and circDLGAP4, a circRNA modulating miR-134–5p, are downregulated in PD. Its upregulation mitigates neuronal damage by inhibiting neurotoxic miRNAs, thus promoting autophagy^[103,104]^. In addition, natural compounds, such as Pramipexole, empagliflozin, and baicalein, have been shown to modulate autophagy by targeting miRNAs in treating PD^[105]^.

### Other strategies

LRRK2 is regarded as a promising target for developing drugs for PD treatment. Numerous LRRK2-kinase inhibitors have been developed, with some advancing to clinical trials. Currently, new clinical trials are underway to evaluate the efficiency of LRRK2 inhibitors DNL151^[106]^, DNL-201^[107]^, and antisense oligonucleotide BIIB094^[108]^ for treating PD patients.

## CONCLUDING REMARKS AND FUTURE PERSPECTIVES

Despite the growing body of studies enhancing our understanding of autophagy in the pathogenesis and treatment of PD, several issues remain to be addressed. First, autophagy is a dynamic and fine-regulated process crucial for cellular homeostasis, especially in neurodegenerative diseases, including PD. The autophagic flux must be considered a whole process rather than up- or downregulating one specific autophagy-related gene. Excessive or uncontrolled activation of autophagy may impair lysosomal activity, inducing autophagy-dependent cell death^[109]^. Monitoring autophagic flux dynamically, rather than only activating or inhibiting specific points, is crucial to prevent overwhelming autophagy activation. Future treatments should aim to restore autophagic balance by targeting the clearance of accumulated substrates and recycling cellular components. Second, a more detailed analysis of autophagy alteration, focusing on specific cell types or cellular compartments rather than whole bulk tissue analysis, will provide a more precise understanding of autophagy changes in PD. For instance, DAT-cre or Cx3cr1-cre mice can be utilized to examine the role of specific autophagy-related genes in dopaminergic neurons or microglia. Transgenic mice labeled with specific organelles, such as mito-QC mice^[110]^, provide a valuable tool for *in vivo* analysis of altered mitophagy and autophagic flux. Therefore, developing new techniques is necessary for investigating autophagy in patients and animal models.

Additionally, therapies based on autophagy face several unresolved issues, such as unstable structures, non-specific functions, and potential risk of neurotoxicity. Whether using chemical reagents or miRNAs targeting autophagy, further research is needed to determine how to maintain proper autophagy levels, as regulating autophagy within a safe and effective range is critical for preventing and treating PD. In conclusion, autophagy represents numerous therapeutic potentials for treating PD. However, further research is needed to fully address these potentials regarding the molecular mechanisms regulating cell-specific autophagy and the interactions between autophagy and other cellular pathways.

## Figures and Tables

**Table 1.** Modeling of therapeutic strategies for altering autophagy in PD

Agent	Target	Main effects	Ref.
**Targeting autophagic initiation**			
mTOR dependent pathway			
Rapamycin and its analogues	mTORC1	Enhanced α-syn clearance and protected dopaminergic neuron survival	[75,76]
Metformin	AMPK	AMPK activators; enhanced autophagy and protected dopaminergic neurons	[77]
Resveratrol	AMPK	Increased α-syn clearance	[78]
PREP inhibitor (KYP-2047)	Beclin-1	Decreased oligomeric α-syn; activated autophagy	[79]
Isorhynchophylline	Beclin-1	Increased α-syn clearance/reduced α-syn accumulation	[80]
mTOR-independent pathway			
Lithium	IMPase	Reduced apoptosis and mitochondrial dysfunction; Increased clearance of α-syn	[81]
Sodium valproate	Ins	Rescued mitochondrial dysfunction; protected dopaminergic neuron survival	[105]
Carbamazepine	Ins	Reduced apoptosis and mitochondrial dysfunction	[105]
Trehalose	SLC2A	Enhanced mTOR-independent activation of autophagy and promoted α-syn clearance	[82]
**Enhancing lysosomal function**			
Rifampicin	Lysosome	Restored lysosomal acidification	[83]
clioquinol	Lysosome	Restored lysosomal acidification; increased lysosomal function	[84]
ML-SA1	Lysosome	Increased lysosomal biogenesis and function	[85]
Nilotinib	Lysosome	Blocked c-Abl; modulated lysosomal maturation	[86]
2-Hydroxypropyl-β-cyclodextrin	TFEB	Increased α-syn clearance	[87]
Ambroxol	GCase	Increased GCase activity; restored lysosomal function	[88]
Isofagomine	GCase	Stabilized GCase; increased α-syn clearance, restored lysosomal function	[88]
Acidic nanoparticles	Lysosome	Lower lysosomal pH; stimulated lysosomal degradation; reverted lysosomal dysfunction	[89]
**Targeting mitophagy**			
PINK1/Parkin dependent pathway			
KTP	PINK1	Mimics ATP; enhanced PINK1 activity and PINK1-mediate mitophagy	[92]
niclosamide	PINK1	Induced mitophagy by causing mitochondrial depolarization	[93]
FT3967385/MF-094	Parkin	USP30 inhibitors; induced mitophagy by blocking the deubiquitination process that opposes Parkin	[94]
PINK1/Parkin independent pathway			
deferiprone	Iron chelation	Induced mitophagy independently of PINK1/Parkin	[97]
AUTACs	Unknown	Mediated ubiquitin-dependent mitophagy but not PINK1/Parkin dependent	[98]
**Regulating autophagy by miRNAs**			
lncRNA OIP5-AS1	miR-137	Inhibited miR-137; promoted mitochondrial autophagy and protected neurons from degeneration	[103]
circDLGAP4	miR-134-5p	Inhibited miR-134-5p; enhanced autophagy, reduced apoptosis, and decreased mitochondrial damage	[104]
Pramipexole	miR 7	Inhibited miR 7 and actived autophagy	[105]
Empagliflozin	miR 211 5p	Upregulated Beclin 1 mediated autophagy, executed neuroprotection	[105]
Baicalein	miR 30b	Inhibited miR 30b; induced mitochondrial autophagy via BNIP3/Nix pathway	[105]
**Other strategies**			
DNL151/DNL-201	LRRK2	LRRK2 inhibitors	[106,107]
BIIB094	LRRK2	Antisense oligonucleotide targeting LRRK2	[108]

PD: Parkinson’s disease; α-syn: α-synuclein; TFEB: transcription factor EB; PINK1: PTEN-induced kinase 1; KTP: kinetin triphosphate; AUTACs: autophagy-targeting chimeras; LRRK2: leucine-rich repeat sequence kinase 2.
